# Neurocognitive basis of model-based decision making and its metacontrol in childhood^☆^

**DOI:** 10.1016/j.dcn.2023.101269

**Published:** 2023-06-16

**Authors:** C.R. Smid, K. Ganesan, A. Thompson, R. Cañigueral, S. Veselic, J. Royer, W. Kool, T.U. Hauser, B. Bernhardt, N. Steinbeis

**Affiliations:** a Department of Psychology and Language Sciences, University College London, United Kingdom; b Clinical and Movement Neurosciences, Department of Motor Neuroscience, University College London, United Kingdom; c Wellcome Centre for Human Neuroimaging, University College London, United Kingdom; d McConnell Brain Imaging Centre, Montreal Neurological Institute, McGill University, Montreal, Canada; e Department of Psychological & Brain Sciences, Washington University in St. Louis, St. Louis, MO, United States; f Max Planck University College London Centre for Computational Psychiatry and Ageing Research, United Kingdom

**Keywords:** Model-based decision-making, Metacontrol, Childhood, Reinforcement learning, Cortical thickness

## Abstract

Human behavior is supported by both goal-directed (model-based) and habitual (model-free) decision-making, each differing in its flexibility, accuracy, and computational cost. The arbitration between habitual and goal-directed systems is thought to be regulated by a process known as metacontrol. However, how these systems emerge and develop remains poorly understood. Recently, we found that while children between 5 and 11 years displayed robust signatures of model-based decision-making, which increased during this developmental period, there were substantial individual differences in the display of metacontrol. Here, we inspect the neurocognitive basis of model-based decision-making and metacontrol in childhood and focus this investigation on executive functions, fluid reasoning, and brain structure. A total of 69 participants between the ages of 6–13 completed a two-step decision-making task and an extensive behavioral test battery. A subset of 44 participants also completed a structural magnetic resonance imaging scan. We find that individual differences in metacontrol are specifically associated with performance on an inhibition task and individual differences in thickness of dorsolateral prefrontal, temporal, and superior-parietal cortices. These brain regions likely reflect the involvement of cognitive processes crucial to metacontrol, such as cognitive control and contextual processing.

## 1 Introduction

To engage in optimal decision-making, individuals need to link their actions to associated outcomes. Classical learning paradigms propose that this challenge is solved by means of two distinct systems that differ in their flexibility and computational cost, with one operating habitually and the other in a more goal-directed fashion (Boureau et al., 2015, Daw, 2018, Daw et al., 2005). Habitual and goal-directed strategies have been formalized in model-free and model-based reinforcement learning algorithms (Daw et al., 2005; Dolan & Dayan, 2013; Gläscher et al., 2010). Whereas the former engenders value-based learning and relies predominantly on tying actions to previous rewards, the latter relies on using an internalized model of the world, matching the rewards attained with the appropriate actions depending on the context (Daw et al., 2011, Kool et al., 2016).

Model-free decision-making is not always adequate but is cognitively less costly as it draws on cached values of past actions. On the other hand, model-based decision-making is more accurate and costly, as new values must continuously be computed (Keramati et al., 2011). Furthermore, optimally responding to different environmental demands, with the inherent processing limits of human cognition, requires dynamic arbitration between the costs and benefits of both decision-making systems (Dubois et al., 2022, Lieder and Griffiths, 2019). For example, for everyday tasks, the efficiency of habitual decision-making might be preferred and allows saving of cognitive resources, while to be successful in novel or complex scenarios, more goal-directed methods may be required. Human decision-making, therefore, continuously requires the arbitration of the potential rewards and costs associated with each action (Bolenz et al., 2019, Boureau et al., 2015, Ruel et al., 2021), a process known as *metacontrol*.

Prior work found that the display of model-based decision-making emerged only in adolescence and increased through adulthood when using decision-making tasks originally designed for adults (Decker et al., 2016, Nussenbaum et al., 2020, Palminteri et al., 2016, Potter et al., 2017). Recently, it has been shown that children as young as five displayed model-based decision-making and that its use continuously increased throughout development (Smid et al., 2022). Importantly, the dynamic deployment of these model-based vs. model-free systems appears to emerge later in life. By manipulating the amount of reward one could gain, we previously showed that adults dynamically increase their model-based reasoning for bigger rewards (Kool et al., 2017), a process termed metacontrol. In contrast to adults, children did not reliably display such metacontrol (Smid et al., 2022), but instead showed substantial individual differences. While it is reasonable to assume that metacontrol emerges reliably during adolescence (Insel et al., 2017), in the current study we focus on examining individual differences in both model-based decision-making as well as metacontrol and study what supports the emergence of these abilities during childhood.

Correlational evidence and experimental manipulations suggest that working memory and inhibition are relevant to model-based decision-making in adults and may underlie this process (Otto et al., 2015, Otto et al., 2013, Potter et al., 2017). For example, a study investing cognitive correlates of model-based decision-making measured with the Daw task in adults found a relationship between higher model-based decision-making and better processing speed and working memory, which they interpreted as a better ability to compute more possibilities for a model-based system (Schad et al., 2014). Thus, model-based control might be related to individual differences in the ability to manipulate complex sequence information, hence making it easier for some to compute model-based predictions. Further, in a sample of 9–25-year-olds, it was shown that fluid reasoning was linked to model-based decision-making (Potter et al., 2017). It has also previously been suggested that inhibitory control, the ability for internally maintained goals to overcome prepotent or stimulus-driven responses, is linked to the model-based system (Otto et al., 2015). Supporting this, the study found that individuals with a higher hallmark of cognitive control, as measured with the Stroop and AX-CPT tasks, also displayed more model-based decision-making (Otto et al., 2015). In contrast, the neurocognitive foundations of efficient metacontrol are much less studied (Bolenz et al., 2019, Kool et al., 2017, Kool and Botvinick, 2014). While metacontrol appears to be present during adolescence, increases into adulthood (Bolenz and Eppinger, 2021), and decreases in older age (Bolenz et al., 2019), its cognitive underpinnings are unclear. However, it has been proposed that executive functions might be relevant (Davidow et al., 2018, Dezfouli and Balleine, 2013, Keramati et al., 2011, Keramati et al., 2016; Lee and Keramati, 2017; Miller et al., 2018; Otto, Gershman et al., 2013). Cognitive abilities could be relevant in several ways in the successful arbitration between model-free and model-based systems. For example, Keramati et al., 2011 suggested that arbitration is determined by the value of information and reflects tradeoffs between speed and accuracy. On the other hand, the link between cognitive abilities and model-based decision-making may be related to a general aspect of intelligence, referring to the ability to divide complex tasks into larger chunks, making them easier to process (Bhandari and Duncan, 2014). To illuminate how cognitive abilities in the form of executive functions, fluid reasoning, and crystallized intelligence may be related to model-based decision-making and its metacontrol, we ran an extensive battery of executive function tasks in the current study.

Prior work on the neural underpinnings of model-free and model-based decision-making has sought to uncover distinct signatures of associated prediction errors. Some studies suggest distinct regions for model-based decision prediction errors, such as the posterior parietal cortex (O’Doherty et al., 2015), the dorsomedial prefrontal cortex (PFC) (Doll et al., 2015), and the (dorso) lateral prefrontal cortex (DLPFC) in particular (Beierholm et al., 2011, Cremer et al., 2021, Doll et al., 2015, Gläscher et al., 2010; Lee et al., 2014; Smittenaar et al., 2013), while model-free prediction errors have been mainly localized to the (ventral) striatum (Beierholm et al., 2011, Gläscher et al., 2010, O’Doherty et al., 2015) or the putamen (Doll et al., 2015, but see also Daw et al., 2011; Sanfey and Chang, 2008). A potential causal role of the DLPFC in model-based decision-making was identified via direct manipulation of the DLPFC via TMS, which led to a reduction in model-based decision-making (Smittenaar et al., 2013).

In contrast, only a few studies have addressed the neural correlates of metacontrol concerning switching between decision-making strategies (Lee et al., 2014; O’Doherty et al., 2015). For example, O'Doherty et al. suggested that the arbitration between model-free and model-based systems was encoded by bilateral inferior lateral PFC, the right frontopolar cortex, and the rostral anterior cingulate cortex (O’Doherty et al., 2015). Meanwhile, Lee et al. found that the arbitration between habitual and goal-directed systems depended on activity in the bilateral lateral PFC (Lee et al., 2014). In addition, a study on adolescents found that the selective upregulation of cognitive control for trials with greater reward in contrast to trials with lesser reward was governed by frontostriatal connectivity (Insel et al., 2017). This could lead to a similar relationship in the context of stake-based metacontrol used in the current study. Taken together, findings from these studies suggest that DLPFC, in particular, may be implicated in both model-based decision-making and its metacontrol, however, presumably serving different respective functions.

These previous studies investigated the relationships between task-related brain activity and decision-making strategies. However, brain activity may be highly susceptible to variability (Faisal et al., 2008; Stein et al., 2005), with increases in noise being specially observed in developmental populations (MacDonald et al., 2009). In addition, the test-retest reliability of individual differences derived from task-related fMRI is low (Korucuoglu et al., 2021), while cortical thickness has been found to be more robust (Velázquez et al., 2021). While previous studies provide insight into the localization of the relevant decision-making processes in the brain with the DLPFC being the most prominent, structural brain correlates such as cortical thickness may be a preferred method to assess how individual differences may relate to cognitive functioning, especially in developmental samples (Ducharme et al., 2021; Fjell et al., 2015; Karama et al., 2011; Tamnes, Ostby, Fjell et al., 2010, Tamnes et al., 2010).

Cortical measures as obtained via MRI are indirect measures of a complex architecture of glia, vasculature, and neurons with dendritic and synaptic processes, which may be closely linked to cognition (Gogtay et al., 2004, Narr et al., 2007). Supporting this, variation within cortical thickness has been previously linked to executive function ability in development (Brito et al., 2017, Krogsrud et al., 2021, Piccolo et al., 2019, Squeglia et al., 2013, Tamnes et al., 2010, Wilke et al., 2003). While cortical thickness patterns vary between individuals and have been shown to be linked to genetic variation (Fjell et al., 2015, Joshi et al., 2011, Panizzon et al., 2009, Rimol et al., 2010), general developmental trends suggest that the cortex thins with age from childhood to adulthood, which has been linked to increased synaptic pruning during adolescence and early adulthood, with grey matter volume peaking in childhood (Giedd et al., 1999, Gogtay et al., 2004, Lenroot et al., 2007, Mills et al., 2016, Wierenga et al., 2014). However, trajectories of maturational and aging effects vary considerably over the cortex, with cortices that are known to myelinate early, such as visual and auditory cortices, showing a more linear pattern of aging compared to frontal and parietal neocortices, which have a more protracted maturational trajectory and continue myelination until adulthood (Sowell et al., 2003). In addition, the relationships between structural brain development and cognition have been variable (Shaw et al., 2006). While some studies show improvements in executive functions and decision-making to be related to cortical thinning during childhood, (Brito et al., 2017, Kharitonova et al., 2013, Squeglia et al., 2013, Tamnes et al., 2010), others have shown the reverse (Schnack et al., 2015). Further, it has been shown that skill acquisition leads to increased thickness in supporting brain regions across the lifespan across multiple domains of cognitive function (Draganski et al., 2004, Engvig et al., 2010, Maguire et al., 2000, Mechelli et al., 2004) The relationship between cortical thickness and cognitive processes is therefore complex and subject to both maturation as well as learning and skill acquisition.

In the current study, we used cortical thickness as a marker of brain structure and linked these to model-based decision-making and metacontrol. We hypothesized that individual differences in model-based decision-making and metacontrol will be reflected in differences in brain structure as indicated by cortical thickness measures. To do so, we employed two methods of assessing the potential relationship with cortical thickness; (1) an exploratory whole-brain analysis and (2) an ROI analysis of the bilateral DLPFC to see if age-independent differences in brain anatomy in 6–13-year-old children are related to model-based decision making and metacontrol.

In sum, this study aimed to investigate the neurocognitive underpinnings of model-based decision-making and metacontrol in children aged 6–13. Based on previous literature, we hypothesized that better executive function ability (working memory, inhibition, cognitive flexibility), as well as fluid reasoning, would be positively related to model-based decision-making in the current study. We also hypothesized that metacontrol may be specifically linked to inhibitory control and cognitive flexibility, as it relies on flexible and dynamic arbitration of different decision-making strategies depending on the environment. In terms of structural brain measures, we expected that individual differences in both model-based decision-making and metacontrol would be linked to individual differences in cortical thickness in the DLPFC, without making a prediction of directionality. For our exploratory whole-brain analysis, we expected that if there was a strong relationship between executive functions and model-based decision-making and metacontrol, cortical thickness of areas that were previously linked to executive function ability such as the DLPFC, the anterior cingulate cortex, and the superior parietal lobe, may be implicated. To test these hypotheses, we related model-based decision-making and metacontrol to performance on an extensive task battery comprising several domains of executive functions and fluid reasoning. While we found no behavioral or structural relationships with model-based decision-making, metacontrol was significantly related to individual differences in inhibition and whole-brain cortical thickness of the entorhinal cortex, the superior parietal cortex, and the bilateral DLPFC in an ROI analysis.

## 2 Methods

### 2.1 Participants

Based on the age-related effect of model-based decision-making across the full sample in a previous study (Smid et al., 2022), we ran a power analysis in G*Power (Faul et al., 2007). The current study would need a sample size of N = 53 to reach 90% power (with ɑ = 0.05, correlation rH1 = 0.39, correlation rH0 = 0). A total of 69 (35 female) participants, with a mean age of 8.99 years (SD = 1.57), and an age range from 6.19 to 12.61 years, were recruited from 20 schools in the Greater London area. Participants were excluded from taking part in the MRI if our safety protocol was not satisfied (e.g., metal in the body; claustrophobia). Following this protocol, 6 participants were not able to attempt the MRI scan due to MRI exclusion criteria (too uncomfortable to go into the MRI). In addition, 2 participants were not able to attempt the MRI scan due to a scanner technical issue on the day. Fifty-nine participants attempted an MRI scan, but for 9 participants the scan was terminated due to discomfort in the scanner. Of the 52 participants who completed the structural scan, 6 had to be excluded due to excessive movement. This resulted in a final MRI sample consisting of 44 (25 female) participants with a mean age of 9.37 years (SD = 1.53) and an age range of 6.19 – 12.61 years. The UCL ethics committee approved the study (Protocol number: 12271/001). In accordance with this, written consent was obtained from both parents and children after a description of the study was provided. Behavioral statistics were run in R and behavioral responses were recorded with Matlab for the two-step sequential decision-making task, as well as the Cognitive Flexibility task. All other tasks were run in EPrime. Data collection was conducted in person on Windows (Lenovo) laptops. Participants took part in a larger intervention study that included data collection on three separate occasions. The current data set was collected at the first testing time point.

## 3 Model-based and model-free measures of decision making

Participants completed a sequential decision-making task that allowed the dissociation of different decision-making strategies (Kool et al., 2016, Kool et al., 2017). This task was adapted for a developmental sample and was previously conducted with children of a similar age range (Smid et al., 2022). The images and narrative of the task were previously adapted for developmental populations (Decker et al., 2016).

**Fig. 1:**
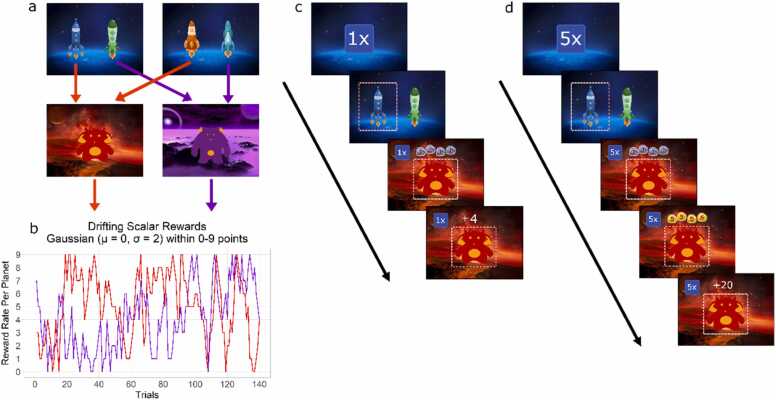
Task Design. a) Schematic of the transition structure with arrows displaying deterministic transitions; if a participant chose the dark blue or the orange spaceship, they would always transition to the red planet. b) At the planets, participants received rewards in the form of space treasure ranging between 0 and 9 pieces according to the drifting reward rate per planet. c) At the start of the trial, participants saw the stake amplifier, which either showed "1x" for low-stake trials or "5x" for high-stake trials. Next, they saw a pair of spaceships and chose one, after which they transitioned to either the red or the purple planet, where they had the opportunity to win pieces of treasure. During low-stake trials, pieces of treasure were displayed in blue with a red "1" on every piece, and participants received points equal to the number of treasure pieces shown. d) During high-stake trials, the blue treasure was displayed first and then, after a delay, turned into gold treasure with a red "5" on top of it, and the number of points received was multiplied by five. Images from (Decker et al., 2016).

Participants were told they were space explorers and that their mission was to collect as much treasure as possible from the two planets (red and purple) they could travel to. Each planet had one alien who gave the participants treasure when they visited their planet. Trials consisted of two stages. Participants saw a pair of spaceships in the first stage and had to choose one spaceship to travel to a planet. There were four spaceships in total, and spaceships were always displayed in the same pairs, of which one spaceship always went to the red planet, and one spaceship always went to the purple planet (see Fig. 1a). In the second stage, participants had to collect treasure from the aliens on the planet. The amount of treasure that could be collected from each planet ranged between 0 and 9 treasure pieces and changed independently throughout the game (see Fig. 1b). Such drifting reward rates have been shown to promote learning and continuous monitoring of rewards won at each planet, in essence allowing a model-based system to capitalize on faster changes in rewards compared to the traditional two-step task (Kool et al., 2016, Smid et al., 2022).

In this task, the difference between a model-based agent and a model-free agent is that a model-based agent can generalize between the spaceships that go to the same planet in each pair, whereas the model-free agent cannot. For example, if the dark blue and the orange spaceship go to the red planet, then a model-based agent should assign the same value to both spaceships. Thus, if a model-based agent chooses the orange spaceship and receives a reward that is higher than expected on the red planet, the value of choosing both the dark blue and the orange spaceship increases, while for a purely model-free agent, only the value of the orange spaceship increases. In short, the model-based agent generalizes reward experiences from one first-stage state (one pair of spaceships) to the other (other pair of spaceships) because they both lead to the same goal (the planet), whereas a model-free agent does not (Doll et al., 2015, Kool et al., 2016).

### 3.1 Metacontrol via stakes manipulation

Low and high-stake trials were introduced to test whether our participants dynamically arbitrate between employing model-free and model-based systems depending on the rewards available. During the trials, participants received rewards in the form of pieces of blue space treasure. On a low-stake trial, the pieces of treasure won directly translated to the number of points won on that trial, e.g., four pieces of blue treasure would have a value of four points (see Fig. 1c). In contrast, during a high-stake trial, rewards were multiplied by five, e.g., four pieces of treasure would increase in value to 20 points. To make this difference between the stakes more salient for the children, on high-stake trials, the treasure turned from blue to gold treasure after a short delay and displayed the number "5" in red on top of the gold treasure pieces, as opposed to “1” on the blue treasure for the low-stake trials (see Fig. 1d). High- and low-stake trials were at an approximate 50/50 ratio and occurred randomly.

Metacontrol was calculated as a difference score in the degree of model-based decision-making during the low- and high-stake trials. The degree of model-based decision-making was measured via a weighting parameter, whereby a value closer to 1 indicated more model-based control, and a value closer to 0 as more model-free control. Using a model with two weighing parameters, one for each stake condition, we measured the difference in the values between the two parameters. A positive value indicated greater model-based decision-making for high-stake trials, and a negative value indicated greater model-based decision-making for low-stake trials. A higher positive value reflects better metacontrol. For further details on the task, the instruction phase, and the computational model, see Smid et al. (2022).

We examined participants’ understanding of the task by asking them to report the deterministic transition structure of the spaceships to the planets after the preparation phase. Understanding of the task structure was high, with 96% of the participants correctly reporting the task structure. Missed trials were excluded from the analysis as participants did not receive rewards on these trials and, therefore, could not learn from them. Participants were excluded if they missed more than 30% of the trials (Smid et al., 2022). On average, children missed only 0.05% of the trials, and the highest percentage of trials missed was 17%. Therefore, no participants were excluded from the analysis.

To assess the relationship between model-based decision-making and metacontrol with age, we fit the dual-systems reinforcement learning model to every participant, and the best-fitting parameters were extracted. Next, we linked these individual best-fitting parameters to age.

### 3.2 Cognitive task battery

An extensive battery of tasks was used to assess a range of executive functions and fluid reasoning and crystallized intelligence (Table 1). This table reflects the main domains for each task, the task name, and the abbreviation for the final included measure in brackets. For the majority of the tasks, trials were either neutral (i.e., congruent trials, stay trials, or go trials) or experimental (incongruent trials, switch trials, or stop trials). The final measures for these tasks capture a difference in performance between the neutral and the experimental trials. For example, the Flanker (Inhib) measure reflects a difference score that measures the speed and accuracy of the incongruent trials and the congruent trials, where a higher value indicates greater processing costs for the incongruent compared to the congruent trials. In short, if this value is positive participants performed worse on the incongruent trials. Additional details regarding the different tasks can be found on the accompanying GitHub page.

**Table 1:** Executive Function and Intelligence tasks.

Domain	Task	Main measure and label
Inhibition	SSRT	Stop-Signal Reaction Time (SSRT) coded inversely: higher values indicate better inhibitory control (SSRT)
	Stroop	Difference between incongruent and congruent trials in composite scores of speed and accuracy (a higher value indicates greater processing costs during incongruent compared to congruent trials; Stroop)
	Flanker (Inhib)	Difference between incongruent and congruent trials in composite scores of speed and accuracy (a higher value indicates worse performance during incongruent compared to congruent trials; Flanker)
	AX-CPT	Difference between the AY and BX trials (PBI Index), where a positive value reflected a higher processing cost on AY trials, indicating more proactive control, and a negative value reflected higher processing cost on BX trials, indicating more reactive control; (AX-CPT)
Cognitive Flexibility	Task-switching	Difference between switch and stay trials in composite scores of speed and accuracy (a higher value indicates greater processing costs during switch compared to stay trials; CogFlex)
	Flanker (Switch)	Difference between switch and stay trials in composite scores of speed and accuracy (a higher value indicates greater processing costs during switch compared to stay trials; Flanker_Switch)
Working Memory	Corsi Block tapping	The highest number of correctly repeated consecutive repetitions referred to here as working memory span (WM_Span)
	N-back	Composite scores for both the 1-back and 2-back conditions. A higher score indicates better working memory performance for each condition. (WM_1back, WM_2back).
Intelligence	Fluid reasoning	Age-standardized measure of fluid reasoning (WASI_Matrix)
	Crystallized intelligence	Age-standardized measure of crystallized intelligence (WASI_Vocab)

### 3.3 MRI acquisition and cortical thickness measurements

High-resolution T1-weighted images were obtained using a Siemens 3.0 Tesla Prisma scanner located at the Birkbeck-UCL Centre for Neuroimaging (BUCNI) equipped with a 32-channel whole-head coil. Images were acquired with the sequence tfl3d1_16ns with a flip angle of 9°. Echo Time was set to 0.00298, and Repetition Time to 2.3. A total of 208 slices with a voxel size of 1x1x1 mm3 were collected, and the acquisition matrix ranged over 256 × 256. To limit head motion, children were requested to keep their heads as still as possible and foam inserts were placed between the head and head coil to ensure the head was snug in the coil. Visual stimuli were projected onto a screen in the magnet boar that could be viewed via a mirror attached to the head coil. Participants watched cartoons without sound during the acquisition of the structural scan.

Structural MRI images were processed with FreeSurfer (Version 6.0.0; http://surfer.nmr.mgh.harvard.edu (Fischl et al., 2002), a software that can label and segment cortex and white matter. After converting the Dicom files to Nifti using dcm2niix, scans were run through FreeSurfer. Then, all scans were visually inspected for quality, and the segmentation was manually corrected in FreeSurfer if not successful. Four independent validators analyzed the scans, and one final validator performed a final inspection of all scans. After corrections, scans were re-segmented using FreeSurfer, until, upon visual inspection, the segmentation quality was adequate, or if it did not reach the final level of acceptance, excluded. Using this method, 44 MRI scans were included, while one scan was left out of further analysis due to excessive movement and poor segmentation. Given the extensive and robust evidence of the causal involvement of DLPFC in model-based decision-making (Beierholm et al., 2011, Cremer et al., 2021, Doll et al., 2015, Gläscher et al., 2010; Lee et al., 2014; Smittenaar et al., 2013), region of interest (ROI) analyses focused exclusively on this area. To create a DLPFC ROI, the Desikan-Killiany atlas was used (Desikan et al., 2006), which is relevant for this age group (Ghosh et al., 2010, Wierenga et al., 2014).

After preprocessing, sulcal and gyral features across individual subjects were aligned by morphing each subject's brain to an average spherical representation that accurately matches cortical thickness measurements across participants, while minimizing metric distortion. For whole-brain analysis, thickness data were smoothed using a 10 mm Gaussian kernel before statistical analysis. Selecting a surface-based kernel reduces measurement noise but preserves the capacity for anatomical localization, as it respects cortical topological features (Bernhardt et al., 2014, Lerch and Evans, 2005). To create the Region of Interest (ROI) of the DLPFC, the Desikan-Killiany atlas was used (Desikan et al., 2006). This atlas allows the automatic division of the cortex into standard gyral-based neuroanatomical regions. This atlas divides the cortex into 34 cortical ROIs in each of the individual hemispheres. We extracted the individual cortical thickness of the ROI that most closely matches the DLPFC in the Desikan-Killiany atlas (ROIs 28 (left) and 64 (right); the Rostral middle frontal cortex) for the ROI analysis.

Cortical thickness data were analyzed using the SurfStat toolbox for Matlab [https://www.math.mcgill.ca/keith/surfstat, (de Waal et al., 2022, Worsley et al., 2009). Cortex-wide linear models were used to assess the effects of age, sex, model-based decision-making, and metacontrol on thickness at each vertex. Findings from the surface-based analyses were controlled for multiple comparisons using random field theory (Bernhardt et al., 2014, Bernhardt et al., 2014, Steinbeis et al., 2012, Worsley et al., 2009). This reduced the chance of reporting a family-wise error (FWE). The cluster-defining threshold was set to p < .01 and the FWE to p < .05. Mediation analysis was conducted in Python using the Pingouin package (Vallat, 2018).

## 4 Results

### 4.1 Markers of model-based decision-making and metacontrol

To assess whether children were sufficiently engaged with and able to perform the task, we compared their performance to chance level. Task performance was calculated as each individual’s corrected reward rate, which reflected the average number of points a participant earned per trial, corrected for each participant's possible rewards based on the drifting reward rates (Fig. 1b). Scores lower than zero indicate performance worse than chance, and scores higher than zero indicate better than chance performance. The mean corrected reward for children was higher than chance (t(68) = 5.10, d = .61, p < .001, 95% CIs [.[0.015034]), and performance was positively correlated with age (r =[T 0.27p =[T 0.02395% CIs [.[0.0448]). This suggests that the children were able to perform the task and that performance improved throughout childhood.

**Fig. 2:**
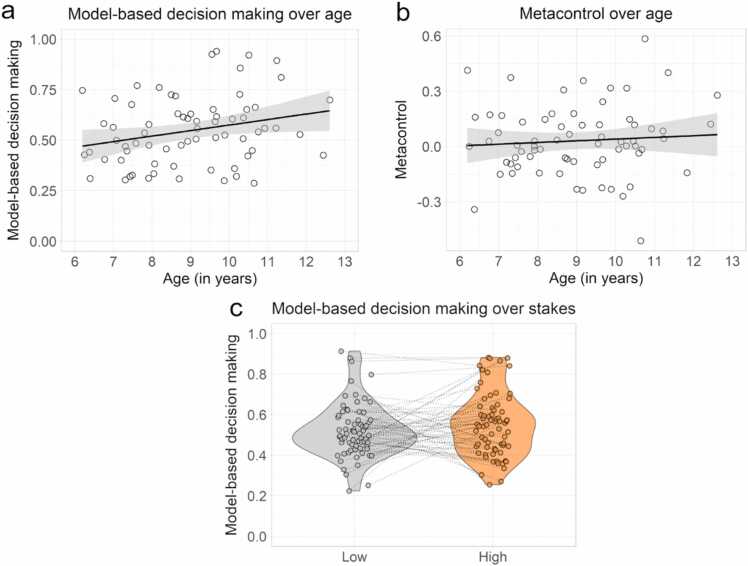
Computational results for model-based decision making and metacontrol. (a) model-based decision making significantly increased with age, (b) while metacontrol did not increase over age, but metacontrol shows substantial individual differences. (c) there was no significant difference in the amount of model-based decision-making over the low- and high-stake trials.

Model-based decision-making was positively correlated to age (r = 0.25, p =[T 0.036see Fig. 2a), and metacontrol was not (r = 0.07, p =[T 0.549see Fig. 2b). There was also no stakes effect for children as a group (t(132.81) = −1.14, p = .255, see Fig. 2c). Neither model-based decision-making (t(65.18) = −1.20, d = −0.29, p = .236), nor metacontrol (t(60.81) = 1.44, d = 0.35, p = .155) differed between sex. Our findings in the current paper thus replicate our previous computational findings in a new sample in childhood (Smid et al., 2022). For additional information on behavioral markers of model-based decision-making and metacontrol can be found on the accompanying GitHub page [https://github.com/ClaireSmid/Neurocognitive_Basis_Metacontrol].

### 4.2 Model-based decision making, metacontrol, and executive functions

**Fig. 3:**
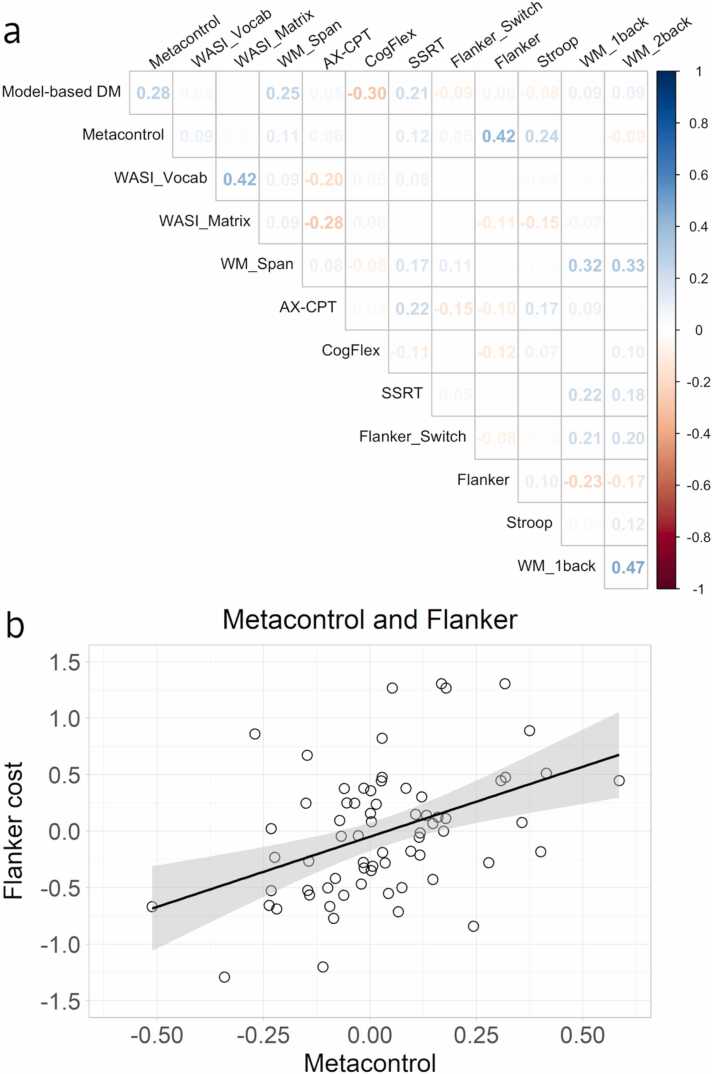
Figures depicting the relationship between model-based decision-making, metacontrol, executive functions, fluid reasoning, and crystallized intelligence. (a) correlation plot of model-based decision-making and metacontrol, executive functions, fluid reasoning, and crystallized intelligence measures. For a list of the measures and their acronyms, see Table 1. The numbers on the plot indicate Pearson’s r values. (b) scatterplot with a linear line indicating the relationship between metacontrol and the Flanker processing costs.

Next, we assessed the relationships between executive functions, model-based decision-making, and metacontrol using simple bivariate correlations (Fig. 3a).

Model-based decision-making was positively correlated with working memory span (r = 0.25, 95% CI [0.01, 0.46], p = .039), indicating that a higher working memory span was correlated to a higher display of model-based decision-making. Model-based decision-making was also negatively correlated to the cognitive flexibility task-switching measure (r = −0.30, 95% CI [−0.50, −0.07], p = .011), which indicates that it was related to lower processing costs during the switch trials, see Table 1.

Metacontrol was positively correlated to the Stroop measure (r = 0.24, 95% CI [−0.004, 0.45], p = .046), indicating that higher processing costs on the incongruent trials on the Stroop task may be related to better metacontrol. Metacontrol was positively correlated with the Flanker measure (r = 0.42, 95% CI [0.21, 0.60], p < .001).

To adjust for multiple comparisons, significance was next adjusted using the Bonferroni procedure for family-wise control (21 tests, threshold at p = .0023). Whereas model-based decision-making did not remain significantly correlated with any measures after correction, metacontrol remained positively correlated with the Flanker measure. Thus, greater metacontrol was related to worse performance in incongruent relative to congruent trials on the Flanker task (Fig. 3b).

### 4.3 Cortical thickness and model-based decision making and metacontrol

Overall mean cortical thickness significantly decreased with age for the sample (T(42) = −2.34, p = .024). There was no significant difference in the mean cortical thickness between male and female participants (F1,42) = .21, p = .647). We assessed the relationship between individual differences in model-based decision-making, metacontrol, and cortical thickness. We ran cortex-wide linear models correcting for age and sex and corrected the p-values with FWE and thresholded for significance at p < .01. We also ran a cortical thickness ROI analysis using the bilateral DLPFC.

**Fig. 4:**
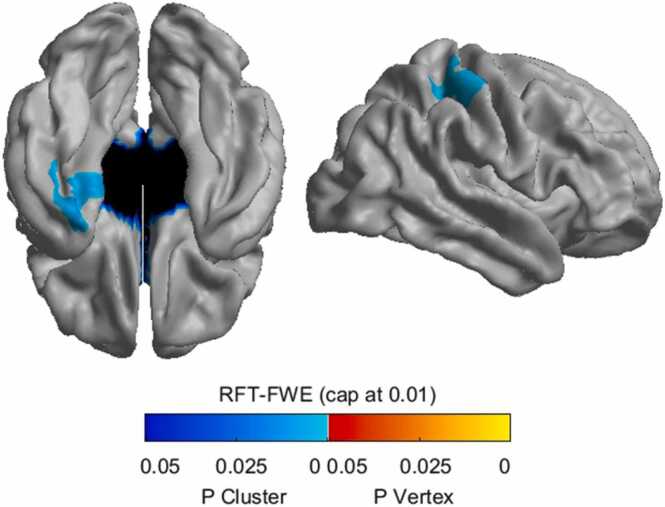
Significant whole-brain clusters of cortical thickness associated with individual differences in metacontrol corrected by age and sex (thresholded at p < .01).

No relationship was found between cortical thickness and indices of model-based decision-making at the whole-brain level. For metacontrol, two clusters survived whole-brain correction (see Fig. 4). Participants with higher metacontrol showed greater cortical thickness in the left temporal lobe encompassing the fusiform gyrus, entorhinal cortex, and parahippocampal gyrus, as well as the right parietal lobe, including the postcentral gyrus and superior parietal cortex, as determined using the Desikan-Killiany atlas (Desikan et al., 2006).

### 4.4 DLPFC ROI analysis

**Fig. 5:**
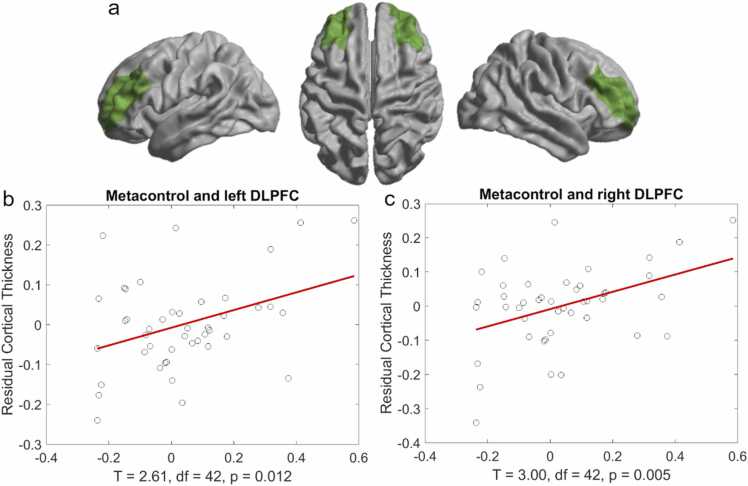
Cortical thickness of the bilateral DLPFC and metacontrol. (a) the ROI for the DLPFC used in the current study is based on the Desikan-Killiany atlas. (b) scatterplot of the relation between metacontrol and residual cortical thickness of the left DLPFC (c) and right DLPFC after correcting for age.

As previous studies have found potential causal links between model-based decision-making and metacontrol and DLPFC (Smittenaar et al., 2013), we also assessed the relationship between cortical thickness in DLPFC bilaterally (Fig. 5a). We ran the ROI analysis with the residual cortical thickness of the DLPFC after controlling for age. As we did not find sex-related differences, we did not control for sex. While we did not find a relationship between thickness in DLPFC and model-based decision-making (p > .09), metacontrol was significantly related to both cortical thickness in left and right DLPFC (T(42) = 2.61, p =[TS82 0.012 T(42) = 3.00, p = .005 respectively; see Fig. 5b and c). These correlations survived Bonferroni correction (threshold at p = .0125). Thus, higher metacontrol was significantly correlated to increased cortical thickness in the bilateral DLPFC for 6–12-year-old children.

### 4.5 A potentially mediating effect of flanker on metacontrol and cortical thickness

**Fig. 6:**
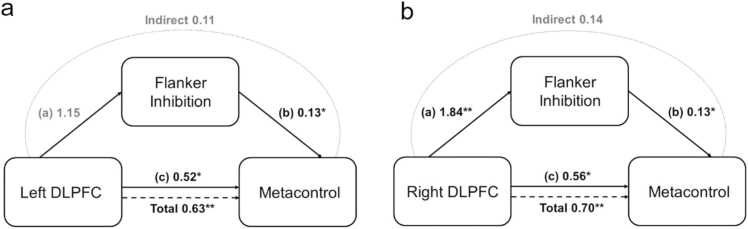
Mediation analysis of the effect of inhibition on the relationship between DLPFC cortical thickness and metacontrol. (a) Mediation model for the left DLPFC, (b) and the right DLPFC. Cortical thickness entered into the mediation analysis was the residual cortical thickness after correcting for age. Asterisks indicate significance (*p* < .05 *, *p* < .01 **, *p* < .001 ***).

Finally, we investigated whether the Flanker measure mediated the relationship between cortical thickness of the bilateral DLPFC and metacontrol. To assess this, we performed a mediation analysis with the Flanker measure as the potential mediating pathway between cortical thickness and metacontrol. For neither the left (indirect: beta = 0.11, se = 0.09, p = .200, 95% CI [−0.02, 0.24], see Fig. 6a), nor the right DLPFC (indirect: beta = 0.14, se = 0.13, p = .284, 95% CI [−0.10, 0.43], see Fig. 6b) was there a mediating effect of Flanker on metacontrol.

We also assessed whether model fit of the whole-brain cortical thickness analysis was improved by adding Flanker as a term, however, it did not improve the model fit.

## 5 Discussion

The current study set out to investigate the neurocognitive basis of model-based decision-making and metacontrol in 6–13-year-old children. To this end, we assessed the relationship of an extensive battery of executive functions, fluid reasoning, and crystallized intelligence as well as brain structure, and related these to indices of model-based decision-making and metacontrol. While we find that model-based decision-making did not show any specific relationships with the executive function task battery or cortical thickness measures, metacontrol showed a specific relationship with performance on an inhibition measure and cortical thickness in temporal, superior parietal, and prefrontal brain regions.

We report a relationship between metacontrol and performance on the Flanker task. Specifically, we found that better metacontrol was related to worse performance in the incongruent trials than in the congruent trials on the Flanker task. Previous work has shown that the preferential allocation of cognitive resources is in part driven by frontostriatal connectivity (Insel et al., 2017) and that considerations of allocating cognitive effort are, in turn, linked to indices of cognitive control (Kool et al., 2017, Kool et al., 2018, Kool and Botvinick, 2014). As such, our findings appear contradictory to this, where greater metacontrol is linked to less inhibitory control. An alternative explanation is that our measure of inhibition might sensitively index the avoidance of effort, as reflected by worse performance on difficult trials. Given that metacontrol entails the reward-sensitive allocation of cognitive effort, such a relationship would make sense. Thus, metacontrol may reflect the ability to engage in successful effort avoidance, an ability that undergoes substantial changes in middle childhood (Niebaum et al., 2019, Niebaum et al., 2020). As higher-order processes, both model-based decision-making and metacontrol are executed because of the concerted functioning of a multitude of cognitive and motivational processes that undergo different developmental trajectories. As a result, we believe it is unlikely for the same set of processes to account for individual differences across all age groups in equal measure. Future work is required to examine this in closer detail.

While it is difficult to say with certainty that worse performance on an inhibition task reflects the avoidance of effort, several pieces of evidence support such an interpretation. First, it has been shown that children as young as 6 years are indeed sensitive to cognitive effort and make choices (Chevalier, 2018, Ganesan and Steinbeis, 2021). Second, a similar relationship, albeit not significant, can be seen with performance on the Stroop task in the present study. Thus, children with higher metacontrol might have displayed similar sensitivity to task-related effort, something that is presumably measured better by tasks that have both high and low-effort components (i.e. incongruent and congruent trials), as opposed to measuring executive performance per se (Lieder and Griffiths, 2019, Niebaum et al., 2019, Niebaum et al., 2020, Ruel et al., 2021). Lastly, as we did not find strong relationships between improved other executive functions and metacontrol, this could indicate that metacontrol may not be supported by executive functions as we measured them in our current study, namely in the three subdomains of working memory, cognitive flexibility, and inhibition. The current results suggest that selective monitoring of effort and reward associated with specific actions in response to the environment is involved, and that, linking metacontrol to successful contextual monitoring and effort avoidance on other tasks could provide evidence for this theory.

In an exploratory whole-brain analysis, we found that individual differences in metacontrol were significantly related to two distinct clusters, one in the left temporal lobe and one in the right superior parietal cortex. The temporal lobe cluster spanned areas involved with memory (Druzgal and D’Esposito, 2001, Jessen et al., 2006, Mion et al., 2010, Rodrigue and Raz, 2004), as well as contextual learning (Aminoff et al., 2007, Coutureau and di Scala, 2009, Peng and Burwell, 2021). The superior parietal lobe cluster spanned areas that have previously been linked to working memory (Koenigs et al., 2009), cognitive control (Loose et al., 2017), and planning (Randerath et al., 2017). Thus, these clusters span brain regions previously implicated in cognitive abilities relevant to metacontrol. Contextual-based learning is relevant as metacontrol in the current study represents the ability to increase computationally effortful performance when beneficial selectively. In addition, the previous link between the superior parietal cortex with cognitive control and planning is relevant, as active prioritization of when to employ model-based decision-making across contexts relies on being able to control when to use which decision-making strategy and selectively switching between them based on context. Using an ROI analysis, we found that the cortical thickness of the bilateral DLPFC was positively related to increased metacontrol, a brain region that has previously been found to be involved in cognitive control and computationally effortful decision-making strategies (Beierholm et al., 2011, Cremer et al., 2021, Doll et al., 2015, Gläscher et al., 2010; Lee et al., 2014; O’Doherty et al., 2015; Smittenaar et al., 2013). Attempts to integrate behavioral and neural measures to account for metacontrol suggest that these account for distinct portions of variance and constitute unique effects. Further work will be required to delineate the computational function of these brain regions in the context of actual task performance.

Given the previous evidence of a relationship between model-based decision-making and executive functions, fluid reasoning, and crystallized intelligence (Otto et al., 2015, Otto et al., 2017, Otto et al., 2013, Potter et al., 2017), the absence of such links in the present sample was surprising. Indeed, we hypothesized that the same executive functions that have been linked to model-based decision-making and metacontrol in adulthood would support this in childhood. At the very least, this finding suggests that the relationship between model-based decision-making and performance on executive function tasks is not straightforward, particularly in the absence of information on how effortful and motivating children might have found the executive function tasks. Surprisingly, and similarly to the behavioral analyses, neither whole-brain nor ROI analyses point to any specific relationships with model-based decision-making in our study. We collected similar measures as used in prior work, reporting significant relationships with model-based decision-making, such as working memory, fluid reasoning, and cognitive control. Differences between prior studies (Potter et al., 2017, Otto et al., 2013, Otto et al., 2015) and our findings presumably relate to differences in measures and samples. In the current study, the absence of a wide age range makes it more speculative over whether the same or different processes underpin model-based decision-making and metacontrol at different developmental time points. A critical difference between the current and previous studies relates to the task used to measure model-based decision-making. Previous studies relied on the traditional two-step task which uses stochastic transitions and, compared to the presently used two-step task with a deterministic task structure, was complex and cognitively more demanding. In essence, it is simpler to employ model-based decision-making on the current task, and a higher degree of model-based decision-making is, in turn, coupled with larger rewards (Kool et al., 2016, Kool et al., 2017). It may well be that prior findings of associations with model-based decision-making and performance on cognitively taxing tasks might be related to task complexity, as opposed to true underlying relationships. It should also be noted that correlating task performance indicates associations at the individual difference level and, not necessarily, whether these processes are used in the context of complex decision-making tasks.

Prior work has described human brain development as a non-linear process, both structurally and functionally, with regional fluctuations in thickness and density during childhood and adolescence (Giedd et al., 1999, Gogtay et al., 2004, Johnson, 2001, Lenroot et al., 2007, Mills et al., 2016, Sowell et al., 2003, Thatcher, 1992). While developmental trajectories in cortical thickness measures from childhood to adulthood show a loss of thickness and density with age, linked to increased synaptic pruning during adolescence and early adulthood, the speed of cortical thinning and the regionality can differ between individuals, potentially mediated by genetic and environmental factors (Fjell et al., 2015, Joshi et al., 2011, Panizzon et al., 2009, Rimol et al., 2010, Shaw et al., 2006). Previous studies have linked greater cortical thinning in development to better executive function ability (Piccolo et al., 2019, Squeglia et al., 2013) and to intelligence (Karama et al., 2011, Narr et al., 2007). Crucially, longitudinal studies have demonstrated that the trajectory of changes in cortical thickness, rather than the absolute thickness at any time point, appears to be most closely related to cognitive outcomes (Fjell et al., 2015, Giedd et al., 1999, Mills et al., 2016, Shaw et al., 2006, Wierenga et al., 2014). The absence of longitudinal data precludes the ability to speak to how brain development might have shaped individual differences in metacontrol. It is known that prefrontal cortical brain regions are among the latest to mature (Bethlehem et al., 2022, Gogtay et al., 2004, Sowell et al., 2003) and thus we conclude that late-developing brain regions like DLPFC, in concert with parietal and temporal regions, appear to contribute to higher order cognitive decision-making processes like metacontrol during childhood.

While we did not find strong relationships between metacontrol and executive functions in the current study, we did find a significant relationship with effort avoidance on the Flanker task. Previous studies investigating effort avoidance in middle childhood have suggested that the contextual monitoring of environmental demands and the adaptive response to changes in effort in absence of reward is an ability that undergoes developmental changes in middle childhood (Chevalier, 2018, Niebaum et al., 2019, Niebaum et al., 2020). From ages 5–12 years, children become progressively more able to indicate awareness of changes in demand in the environment, and more able to respond to them. In this study, we find that higher metacontrol, or the ability to selectively increase model-based decision-making for the highest rewarding trials, is also linked to effort avoidance on the Flanker task, by prioritizing effort for the easier congruent trials rather than the more difficult incongruent trials. It has been shown that both the DLPFC and the anterior cingulate cortex are particularly sensitive to cognitive effort computations (Chong et al., 2017). Previous research has also linked improved demand awareness and the ability to respond adaptively to maturation in the (lateral) prefrontal cortex and the dorsal anterior cingulate (Chong et al., 2017, Niebaum et al., 2019, Niebaum et al., 2020). Thus, the metacontrol measure, in contrast to the model-based decision-making measure, may be reflecting the successful monitoring of task demands as well as the ability to act on these shifts in the environment. Rather, the model-based decision-making measure on its own does not reflect this adaptive response to the environment, and instead, reflects how much participants utilize the internal structure of the task when planning their next decision. In the exploratory whole-brain analysis, we found significant clusters in the parahippocampal cortex and superior parietal cortex, areas that have been linked to contextual learning and planning, which provide support for this hypothesis (Aminoff et al., 2007, Coutureau and di Scala, 2009, Peng and Burwell, 2021, Randerath et al., 2017). We hypothesize that metacontrol revolves around the flexible and adaptive arbitration of cost-benefit calculations, reflecting proactive control around decision-making. This ability would in turn be linked to successful effort avoidance when there is no benefit to engaging in increased effort (Niebaum et al., 2019, Niebaum et al., 2020). The ability to display effort avoidance and selective engagement of effort for more reward has previously been shown to undergo changes during middle childhood, and to be linked to cortical maturation, particularly in prefrontal areas (Niebaum et al., 2019, Niebaum et al., 2020). Therefore, we believe that this is reflected in the relationship between increased cortical thickness in the DLPFC in the ROI analysis, as well as areas in the superior parietal cortex and temporal lobe which have previously been linked to contextual learning and memory. Future work should include tasks sensitive to measuring effort avoidance to further support this hypothesis.

This study has several limitations. While the MRI sample used in the current study (N = 44) is relatively large compared to typical developmental neuroimaging studies, it has been recently suggested that sampling errors could drive significant associations and that robust effects will require sample sizes of the order of hundreds or thousands of participants depending on the phenotype in question (Marek et al., 2022). Thus, the current study may have an underpowered sample, and the results should be interpreted with caution. In the ROI analysis in the present study, the significant association with cortical thickness in an a priori region of interest lends greater credence to the results, as this area was defined based on previous literature and not via a non-independent cluster. It also must be noted that all our measures are correlational. Future research on model-based decision-making and metacontrol in development may wish to adopt experimental manipulations *(*i.e., dual-task paradigms), ideally in a longitudinal setting, to draw stronger inferences, particularly about the role of executive functions. In addition, a longitudinal study, as opposed to the current cross-sectional study, would allow inference of how intra-individual variability in performance on executive function and decision-making tasks would allow insight into how these may be related (Cañigueral et al., 2022). In addition, further research into the development of effort avoidance in relation to metacontrol would allow clarification on which processes support the adaptive prioritization and selective application of effort in development. A previous study found that connectivity between the striatum and the prefrontal cortex mediated the display of titration of cognitive performance according to environmental demands in adolescents; finding that the connectivity selectively increases during high stakes and with age across adolescence (Insel et al., 2017). Thus, the late development of corticostriatal connectivity may be a key development related to improvements in metacontrol via optimal goal-directed behavior. Finally, the findings in the current study are limited to anatomical findings and therefore cannot speak to functional processes. Future work using multimodal imaging of structural and functional brain indices could further illuminate their interrelationship and our understanding of their respective and combined functional role.

In sum, the current study set out to investigate the underlying neurocognitive basis of model-based decision-making and metacontrol. We could not replicate previously reported relationships between model-based decision-making and executive functions, nor any links with markers of brain structure. However, we found that metacontrol was linked to worse performance on inhibition trials and to increased cortical thickness in the temporal and superior parietal lobe and the DLPFC. Metacontrol reflects the optimal use of limited cognitive resources, and our findings suggest that during childhood, this is supported by several brain regions linked to contextual learning and cognitive control. Further, our results suggest that the relationship between model-based decision-making and other cognitive functions is presumably task-dependent. More extensive investigation with a larger battery of tests, bigger samples, and a better characterization of task-specific associations with goals and effort should illuminate how sophisticated value-based decision-making strategies change during development.

## Ethical approval

Ethical approval for this study was obtained from our university’s research ethics committee in compliance with national regulations, project ID number: 12271/003.

## CRediT authorship contribution statement

C.R.S. conceived, designed, and performed the experiments and analyzed the data. K. G. and A. T. helped conceive the experiments and collect the data. J. R. and B. B. helped conceive the neuroimaging analyses, and W. K., T. U. H., the computational modeling. S. V. and R. C. helped revise and write the draft and analyses. N.S. conceived and designed the experiments. All authors read and approved the final version of the manuscript before submission.

## Declaration of Competing Interest

The authors declare the following financial interests/personal relationships which may be considered as potential competing interests: Co-author Nikolaus Steinbeis is an editor for the FLUX special issue at Developmental Cognitive Neuroscience..

## Data Availability

The data and code that support the findings of this study are openly available on Github: https://github.com/ClaireSmid/Neurocognitive_Underpinnings_Metacontrol.
